# Supporting communication in semantic dementia: clinical consensus from expert practitioners

**DOI:** 10.1108/QAOA-08-2014-0016

**Published:** 2015-09-14

**Authors:** Jacqueline Kindell, Karen Sage, Madeline Cruice

**Affiliations:** School of Nursing, Social Work and Midwifery, University of Manchester, Manchester, UK; Department of Health and Applied Sciences, University of the West of England, Bristol, UK; School of Health Sciences, City University London, London, UK

**Keywords:** Delphi, Clinical practice, Frontotemporal dementia, Nominal group technique, Primary progressive aphasia, Semantic dementia

## Abstract

**Purpose:**

The purpose of this paper is to gain consensus regarding the clinical priorities and tasks required in supporting communication needs in those living with semantic dementia and their families, by specialist speech and language therapists (SLTs), working in clinical practice within dementia care settings in the UK.

**Design/methodology/approach:**

A nominal group technique was used, followed by further exploration and refinement of issues using a modified Delphi technique with a group of six SLTs who specialised in dementia care and who had experience of working with individuals with semantic dementia and their families.

**Findings:**

The findings in the study demonstrate a broader scope of practice than is evident within the research literature with this client group. Therapists identified a range of psychosocial issues for both the person with semantic dementia and their family, in particular finding ways to support activity and participation in conversation and explore barriers and facilitators within the communication environment.

**Originality/value:**

This represents the first study to explore everyday practice in this rarer dementia and the information gathered here will be of use to a variety of health and social care professions interested in supporting those with semantic dementia and their families.

## Introduction

Dementia is caused by a number of brain illnesses leading to changes in cognitive functions, behaviour and everyday skills (Dua *et al*., 2012). A less well-known group of dementias is the spectrum of conditions classified as frontotemporal dementias, thought to account for 2-3 per cent of all cases of dementia and a common cause of dementia in those under 65 years of age (Dua *et al*., 2012). These conditions present with a different set of symptoms to more common dementias such as Alzheimer’s disease, in that day-to-day memory and visuospatial skills are relatively preserved, whilst early changes in personality and communication are striking (Neary *et al*., 1998). Semantic dementia is one variant of frontotemporal dementia, where progressive loss of conceptual knowledge (semantic memory) leads to receptive and expressive language difficulties, with individuals experiencing problems in finding words when talking and understanding the speech of others, alongside changes in personality and behaviour (Hodges and Patterson, 2007). Particularly in the North American literature, the condition may also be classified as “the semantic variant of primary progressive aphasia” (Gorno-Tempini *et al*., 2011).

Internationally, a number of centres of expertise have helped develop an understanding of semantic dementia’s atypical presentation and the differential diagnosis of this condition from other dementias (Gorno-Tempini *et al*., 2011; Hodges and Patterson, 2007; Kertesz *et al*., 2010; Neary *et al*., 1998). Following diagnosis, local services provide support and care coordination for individuals with semantic dementia and their families; in the UK, for example, through older people’s mental health services, memory clinics and social care providers. Mental health and memory clinic teams consist of a range of disciplines addressing different needs and issues and aiming to provide holistic support for the person with semantic dementia and their family. Individuals with semantic dementia may be referred to a speech and language therapist (SLT) working within these services for advice about their communication difficulties. This also includes working with family members to advise on appropriate communication strategies at home.

In order to provide high-quality individualised care, health and social care staff consult and synthesise information from a range of sources including research evidence, clinical guidelines and their more experienced peers (Greenhalgh *et al*., 2014). However, when supporting those with semantic dementia and their families, there is little published work to guide practice. Within the UK dementia guideline (National Institute for Health and Clinical Excellence, 2006), frontotemporal dementia is specifically addressed only within differential diagnosis and genetic issues. This lack of guidance is a particular challenge for dementia services which need to provide assessment and individualised intervention for a rarer condition that presents and progresses, in a manner that is different from the standard blueprint of dementia (Kindell *et al*., 2014).

Practitioners must therefore refer to key papers within the research literature. An update of the Carthery-Goulart *et al.* (2013) systematic review examining non-pharmacological interventions in primary progressive aphasia, was carried out for this study (restricted to semantic dementia in this instance), to determine if any recent papers had been published in addition to those identified in the systematic review. Overall, this revealed a relatively narrow scope for interventions in semantic dementia and considerable gaps in evidence, with the area recognised as an emerging area of practice (Taylor *et al*., 2009). Most attention has focused on whether people with semantic dementia can relearn lost vocabulary with some positive results in experimental settings (see Carthery-Goulart *et al*., 2013 for a review of this area and Savage *et al*., 2013 for a more recent study). However, researchers have been unable to ascertain whether this activity improves everyday communication in natural situations and, if so, if this improvement is maintained over time. The use of memory books and picture boards to support participation in conversation has been discussed for those with primary progressive aphasia (Fried-Oken *et al*., 2012; Rogers *et al*., 2000). However, it is unclear whether such approaches are suitable for all types of primary progressive aphasia and in particular, whether there are challenges for their use with those experiencing the underlying conceptual loss that characterises semantic dementia.

There are few studies explicitly targeting everyday skills in semantic dementia. Cartwright and Elliott (2009) worked with four individuals with semantic dementia and their carers in a group situation, using structured television viewing and discussion within sessions. They were able to identify significant improvements in television viewing ability with this format, for example, recalling a greater number of story information units in their discourse post therapy. Bier *et al*. (2011) used structured support to effectively assist an individual with semantic dementia to reacquire a favoured recipe they had stopped cooking. In contrast to the studies described so far, Wong *et al*. (2009) focused their intervention on the spouse of an individual with semantic dementia using a discourse-based intervention to help foster communication using residual abilities, including non-verbal communication, with some promising outcomes.

Lastly it is not clear if, or how, therapies aimed at dementia in general, such as life story work and reminiscence, can be used with this client group. A central aspect to such approaches is to move away from compromised recent memories and to stimulate memories from the long-term past (McKeown *et al.*, 2006; Schweitzer and Bruce, 2008). However, whilst the preservation of long-term memories, in contrast to recent memories, is a typical pattern in Alzheimer’s disease, individuals with semantic dementia often show a reversal of this pattern, with recent memory relatively spared (Hodges and Patterson, 2007). It has, therefore, been suggested that reminiscence may not always be appropriate for people with frontotemporal dementia (Eastern Cognitive Disorders Clinic: Frontotemporal Dementia Toolkit) and that life story work in semantic dementia may need to be adapted to reflect a focus on more recent memories (Kindell *et al*., 2014).

The significant needs of family caregivers of people with frontotemporal dementia have been identified (Nunnemann *et al*., 2012) as well as caregiver education and support (Damianakis *et al*., 2008; Weintraub and Morhardt, 2005; Mioshi *et al*., 2013), though these do not separate out the specific issues families may have when caring for someone with semantic dementia. There is therefore much to learn and share about current and potential therapeutic strategies which support communication needs in individuals with semantic dementia and their families. Thus, this project aimed to explore communication interventions provided to people with semantic dementia and their families by specialist SLTs working in clinical practice within dementia care settings in the UK.

## Method and participants

Participants were recruited from a known expert group, the UK Northern SLT Special Interest Group (SIG) in Old Age Psychiatry (via the first author). The group has been meeting biannually for 25 years and provides a forum for those working in dementia care to review the literature, organise training and explore clinical expertise. The group has a long-standing interest in frontotemporal dementia and semantic dementia and had been discussing practice in this area for several years prior to the study. The authors of this paper worked with this group to build on this work and develop a consensus on practice in this area that would be helpful to a range of health and social care staff working with those with semantic dementia and their families.

A nominal group technique (NGT) was used as the core method in this study. This involves silent generation of ideas; round robin sharing of ideas; group discussions and voting and ranking (Delbecq *et al*., 1975). This was followed by further exploration and refinement of issues using an iterative process adapted from a modified Delphi technique (Delbecq *et al*., 1975). Ten SLTs, who specialised in dementia care and who had clinical experience of working with people with semantic dementia and their families, were identified from the northern UK SLT SIG in Old Age Psychiatry. Invitations were sent and six therapists agreed to participate in the consensus meeting. The therapists had undergraduate degree qualifications in speech and language therapy and two had additional master’s qualifications. The group had extensive experience working as SLTs with an average of 23 years practising (range 16-30 years) and an average of 17 years working with individuals with dementia in both community and inpatient multidisciplinary settings (range 6-26 years). All had experience of working with clients with semantic dementia and therapists had seen a range of between two and 20 clients each with this condition (average 6.5 clients, total 39 people with semantic dementia). The SLTs had worked within health and social care in various ways with individuals with semantic dementia and their families (see Table I), illustrating in-depth and longitudinal experience with this client group and a far broader scope of practice than is currently described in the literature. Given the rarity of semantic dementia, this group provided a unique opportunity to explore clinical practice in this area.

Prior to the meeting therapists were asked to reflect on the cases of semantic dementia they had seen within their practice. The meeting was facilitated by M. Cruice. Following introductions all six independently completed questionnaires that explored their practice in the area of semantic dementia. The purpose of this was to gather background information and to focus participants on the cases of semantic dementia they had seen in their practice before engaging in the four steps of Delbecq’s NGT method as follows:

1. Therapists were asked to silently generate their ideas, reflecting on their own practice and write them down in response to the following question: what aspects are important for the SLT to address when working with people with semantic dementia and their families?
2. Participants then shared one item from their list, using the round robin method until 28 statements were generated.
3. Discussions enabled agreement for three items to be merged together because of their similarity, leaving 25 statements.
4. Participants voted and ranked their top nine priorities. In this way, participants weighted each with a ranking order so that the highest priority was given a ranking of “nine votes”, the next “eight votes”, etc. down to the last given “one vote”. The 25 statements were then put in order according to this ranking.

Lastly, participants reflected on the data generated. The group identified statements that were potentially related and noted that the provision of real life clinical examples to illustrate the statements generated would make them more accessible to a variety of health and social care practitioners. It was agreed that further work would be carried out via e-mail and telephone over the next six months.

The research team (J. Kindell, K. Sage and M. Cruice) then met to examine the 25 statements and used the reflections generated by the group to further revise the data as suggested, including merging overlapping statements. J. Kindell tracked individual clinician’s views using e-mail and telephone calls; logged changes to the drafts; facilitated group e-mail discussion to achieve consensus in a timely manner, and logged examples from clinical practice to describe areas of work. A final set of nine areas of practice was agreed by all members of the group at a final face-to-face meeting, along with practical examples illustrating how each of these areas may be addressed within everyday clinical practice.

## Results

The following nine areas for practice were identified by the group arising out of their experiences in working with this client group:

1. To enable better communication between the person with semantic dementia and their family carer(s) and other professionals, thus creating partnerships in communication.

Therapists reported that an essential part of their practice was to ensure systems were in place so that all involved worked in partnership to share information relevant to the individual’s communication needs. As well as family, friends and neighbours, people with the condition were also in touch with a variety of health, social care, voluntary and private sector workers. Therapist stated that it was important that all involved understood the condition and that ongoing systems were available to share how best to support communication including: regular meetings with all involved taking place in the home; diaries in the home or held by the person with semantic dementia to record important events, issues, topics of conversation or concerns. Partnerships had needed time to grow and develop to ensure communication supports were understood and well used. Therapists felt that this helped support the person with semantic dementia and their caregiver now and in the long term. Part of the on-going role of the therapist was to check systems were working, as well as suggest and reinforce adjustments over time in response to changing needs.

2. Supporting and educating other members of the team about semantic dementia and the person’s particular communication needs to enable them to support the person. This may include joint sessions where the SLT directly facilitates communication between the person with semantic dementia and others such as medical staff, day care staff, etc. to help facilitate their assessments/interventions.

Therapists had found that health, social care and voluntary sector staff often had little experience of semantic dementia and used their knowledge of communication and assessment of the individual concerned, to explain the condition, including how the conceptual loss led to challenges in communication, behaviour and functional skills. Therapists reported that providing large amounts of generic information about semantic dementia, particularly written information, was not always helpful. Information was therefore carefully tailored to be practical in nature, i.e. what does this staff member need to know about this person with semantic dementia for the tasks/activities they need to do. Most often the therapist would meet and discuss the case and, in some instances, undertake joint assessment and therapy sessions. Therapists had worked with psychiatrists, memory clinic nurses, general practitioners, occupational therapists, social workers, day care staff, home care staff, acute hospital doctors and nurses, voluntary sector staff and police mental health liaison officers.

3. The underpinning foundations and values for SLT practice with people with semantic dementia include:
  ▪ A focus on ability within therapy, rather than disability, including strengths with spatial maps, ability to learn and establish a routine, numerical skills, etc.
  ▪ Promoting such skills maintains successful positive experiences; thus maintaining confidence, independence and safety. Multidisciplinary risk assessment may be part of this work in order to maintain everyday skills and abilities including hobbies and activities of daily living.
  ▪ Assessment may include formal and informal methods but must take account of the context of the person and their caregiver’s past and present life, including communication styles and relationships.
  ▪ The SLT may need to be involved over time in order to facilitate ongoing support about communication and continued review in order to avoid a crisis.
  ▪ The care of people with semantic dementia is best carried out using a multidisciplinary team (MDT) approach, where disciplines collaborate to set and evaluate joint goals for therapy and care.

Therapists used person centred dementia care models as foundations for their practice (Brooker, 2007), stressing that using strengths inherent in the individual, was as important as finding out about difficulties. Individuals may, for example, follow a schedule set up between the family and the therapist or engage in activities such as jigsaws, Sudoku, word searches, photography, listening to music, walking or riding a bike and therapists felt that facilitating and sustaining these skills was important in order to maintain quality of life. Risk assessment was highly individualised and carried out in conjunction with other members of the team and care partners. Therapists discussed the importance of positive risk taking along with problem solving to maintain skills where possible. They emphasised the need to focus on communication in everyday life and how this was impacting on the person’s well-being and that of their family. This involved informal assessment methods such as observation and interviews with all concerned, including discussion about changes in personality and communication style.

The length of time therapists were involved with people varied and was determined by the individual’s particular needs at the time, those of their family and local levels of service provision. As needs changed additional information and advice became necessary, so continuity with a service over time enabled proactive, rather than reactive, support and advice. Therapists had found that the rarity of the condition made it harder for families they worked with to access accurate information and appropriate help from services. Whilst therapists thought an MDT approach was important, they reported that input from services varied considerably between, and even within, localities and this was a cause for concern.

4. To provide verbal and written information and advice to the person and their family about semantic dementia and the individual’s particular needs in a timely and appropriate manner. Those involved should be aware of the progressive nature of this condition in order to ensure both a realistic expectation of themselves and of services and to ensure they are prepared both practically and emotionally for future change. This is likely to incorporate not only information about communication but a broader explanation about the role semantics has in an individual’s understanding of the world.

Therapists stated that it was important to provide individualised advice to the person with semantic dementia, where possible, and their family. Therapist reported that helping relatives to understand the relationship between semantics, communication and behavioural change was crucial in order to avoid conflict; for example, understanding that behaviours, or conversation, that might appear “rigid and obsessional” on the surface, arose out of the condition, rather than the person being difficult or self-centred. Therapists spent time identifying the particular issues of concern and then exploring ways to manage that communication issue or behaviour with caregivers. Strategies were discussed in face-to-face sessions and individualised advice sheets were provided which incorporated strategies for practical everyday situations. Areas explored included: receptive and expressive communication difficulties and included modelling appropriate communication strategies; repetitive topics of conversation; family caregiver needs; personality changes; dealing with behavioural change; socially embarrassing communication; and sharing the diagnosis with others. Therapist spoke about the importance of finding out what those involved needed to know about semantic dementia but also, importantly, what they were ready for emotionally, at that particular point in time.

5. To explore meaningful strategies to maintain communicative function.

In their practice therapists explored ways to encourage participation in everyday conversation using compensatory strategies to support communication skills. Such activities included developing word books or key fobs listing the names of people and places; conversation topic books; life story books; scrap books; photo albums or postcard books; writing a daily journal or notebook; standardised shopping lists; visual/photographic supports to facilitate use of appliances, computers, etc.; and prompts by the phone to support conversations.

6. Maintain social contact, engagement and integration for both the couple (person with semantic dementia and their spouse/care partner) and their broader friendship group.

Therapists felt that it was important to recognise that people with semantic dementia and their families had a life outside of the home too. Therapists had observed that communication issues often arose within this arena and there was potential for social isolation if the person or their family began to avoid social situations because of changes with communication or behaviour. Such issues included difficulty engaging in conversation, repetitive behaviour and conversation or topics of conversation not appropriate for the situation at hand. Therapists met with the family and the person with semantic dementia to map social networks and the challenges within them and then to problem solve issues, where possible. Sometimes such meetings included friends whilst, in other instances, written information was provided for those outside the family. Therapists worked with other members of the MDT so that current social contacts and activities could be maintained through additional support, e.g. engaging a support worker to take someone out to play golf with friends.

7. The SLT is likely to engage in a variety of work around current and potential legal, financial, health and social care issues that arise from living with a progressive communication disorder. This includes advocating for the person with semantic dementia where necessary and facilitating the person’s role within such matters wherever possible. Assessment of capacity for such decision-making may be a part of this work.

A number of important personal, health and social care decisions needed the person with semantic dementia to engage through verbal or written communication. This included issues around capacity, consent to treatment, driving, financial issues, care provision, power of attorney, behaviour in the community and contact with the police. Therapists may be involved in this process by assessing capacity or supporting the person’s participation by appropriately simplifying material, using visual materials (photographs and drawings) and/or written prompts, along with utilising preserved areas of understanding in order to explain issues.

8. Working with family and friends to understand the “old” and “new” person with semantic dementia and to help them find acceptance and adjustment, by letting go of the old way of “being” and finding a new way of “being” together that promotes living well with semantic dementia for all involved.

Therapists had observed in their practice that some families naturally adjusted to a new way of being with the person with semantic dementia as their needs changed, while others appeared to struggle more and held on to expectations of the person that they could no longer meet and needed help to work through this. Therapists spoke about the complexities for families in coping with someone who was both the same and different at the same time. Therapists identified practical and psychological aspects to this work such as identifying activities to do together; psychological coping mechanisms; working through feelings of embarrassment; and recognising when extra help was needed and facilitating referrals for this.

9. To introduce the person with semantic dementia and their family to supportive networks in order to help maintain skills/engagement with a view to support quality of life. In some instances this may involve directly providing such opportunities, e.g. providing opportunities for family carers to meet up with other carers to share worries and concerns, learn from each other and for education and support.

Therapists had found in their work that some people with semantic dementia and their families had found it difficult to maintain their prior social life and friendships due to the changes in social communication experienced by the person with semantic dementia or due to the reaction of others. Therapists reported that in these instances, making new friends via dementia groups and support services had become important. Some individuals only needed the therapist to give them a contact number or leaflet whilst, for others, the therapist physically took them to the service and introduced them. Occasionally therapists directly provided these services via small peer group support, e.g. a frontotemporal dementia support group, a primary progressive aphasia support group. Therapists stated that the stage of the individual with semantic dementia and the emotional readiness of the family, were both factors that appeared to influence the choice of group support. For example, one spouse of an individual with early semantic dementia found attending a carers group with carers of people with later stage behavioural variant frontotemporal dementia extremely distressing because of the behavioural difficulties being discussed.

In summary the NGT identified a range of priorities for clinical practice in addressing the communication needs of individuals with semantic dementia and their families. The group in this study recommended a broad approach, delivered in partnership with the person with semantic dementia, their family and friends and formal providers of health and social care. Providing accurate and individualised information about communication needs to all those involved, was stressed, along with the provision of products to support an individual’s communication (e.g. conversation topic books, word books and life story books). Support for care partners also stressed the need to help adaptation to changing needs. Therapists considered issues of social contact and isolation and opportunity for support for both the person with semantic dementia and their care partners. A variety of values underpinned the work of these therapists, for example, the requirement to focus on the person’s abilities, not just their deficits within therapy and the need to promote and maintain skills. These values were in keeping with a person centred dementia care approach (Brooker, 2007).

## Discussion

The International Classification of Functioning (ICF), Disability and Health (WHO, 2001) provides a useful framework to conceptualise therapeutic approaches to management of the communication difficulties seen in semantic dementia and has been explored with respect to communication within dementia care in general (Bryan and Orange, 2005). The ICF outlines a biopsychosocial approach that considers health at the levels of the body, the individual and society, allowing for potential intervention to be delivered at each of these levels. Thus, conditions such as semantic dementia can damage body structures (e.g. brain cells) leading to difficulties with body functions (e.g. mental functions, such as language skills). Research examining communication in semantic dementia has been dominated by approaches aiming to improve mental functions, such as word finding, using experimental word relearning tasks, with therapy focused largely on the individual with semantic dementia (Carthery-Goulart *et al*., 2013).

The value of the ICF for semantic dementia though is in its explicit identification of limitations in executing everyday activities (e.g. conversation), restriction in participation in life situations and barriers and facilitators in the environment including physical, social and attitudinal factors. In contrast to the dominant focus of the research literature, this group of therapists targeted these psychosocial aspects within their practice, i.e. aiming to improve activity, participation and the environmental context, rather than work on specific mental functions such as word finding. Products such as topic books, word books (listing names and other key vocabulary) and life story books aimed to foster participation in personally relevant conversation. Work with family and health and social care staff aimed to develop an understanding of the condition, manage emotional and practical needs and foster appropriate interpersonal support within the communicative environment[1]. Clinical priorities were therefore dictated, not just by the level and nature of the language difficulty, but also broader issues therapists observed within the everyday environment. These findings overlap those of Muo *et al*. (2005) who explored the use of the ICF to describe the health needs of those with Alzheimer’s disease. As in this study, the importance of communication in daily life, social relationships and recreation and leisure were emphasised, with the authors noting that these areas may be missed in many standard scales assessing activities of daily living (Muo *et al*., 2005).

Whilst the research literature in semantic dementia almost exclusively focuses on therapy with the person with semantic dementia, this group of therapists directed their attention to those around the person and on important relationships for communication. This is consistent with a “relationship centred” approach to dementia care which recognises and values the differing perspectives and needs each individual brings to the situation, including the person with dementia, family and staff (Nolan *et al*., 2004). Thus therapy simultaneously targeted the person with semantic dementia, family members and the broader communication network including friends, neighbours, other professionals and paid caregivers. This broad based, yet individualised approach, has not been described in the semantic dementia literature but presents as an important way to deliver practice. By mapping out communication environments, therapists were able to work with immediate family members but also consider communication needs in other situations outside of the home. Such a notion is in keeping with the drive for “dementia friendly communities” (Duggan *et al*., 2008; Mitchell *et al*., 2003) and the need to target social isolation in people with dementia (Alzheimer’s Society, 2013). Therapists in this study worked predominantly on a domiciliary basis, rather than in a clinic situation and this way of working allowed them the freedom to seek out different situations and people, depending on where communication need was identified.

Therapists were concerned about the lack of an agreed pathway and provision for this client group. Whilst the needs of this group overlapped with those of people with other dementias, there were clear differences in terms of behaviour and communication and subsequent support and information needs. Another issue of concern was the large variation in levels of detection of semantic dementia between areas of the country. Therapists identified the requirement for a MDT approach to the care of people with semantic dementia and, in some instances, this need was met and worked well. Therapists gave examples of working closely with occupational therapists to promote communication in everyday activities or with social workers to ensure communication needs were integrated into the plan of care. However, in other instances, provision was fragmented with a lack of understanding, inadequate service provision and a failure to ensure continuity of care. Due to the lack of available provision locally therapists sometimes started their own groups to support people with semantic dementia and their families.

Finally, this group of therapists positioned this condition as a progressive dementia, with a clear sense within their work that whilst they had to deal with the challenges present in the here and now, they also had to plan for future adaptations because of the progressive nature of the condition. This was evident in their information to relatives and in the provision of products to support conversation. Rogers and Alarcon (1998) argue that, when working with those with primary progressive aphasia, the first principle of proactive management should be “anticipatory implementation of treatment goals”, specifically that “therapy goals should be implemented in anticipation of continued decline in communication independence” (p. 645). The work of therapists here is in keeping with this. Therapists also adapted therapies from dementia care, such as life story work. For example, one therapist had developed a *Who*’*s Who and What*’*s What* book, which focused not on past events but on current relationships and activities. In the later stages, work was most often focused on the family but, at all times, placing the individual needs and perspective of the person with semantic dementia at the centre of the process. This has been described generally in dementia as “carer-focused person centred” work (James, 2011).

There is a broader important point here, in terms of representation and positioning of semantic dementia within both the literature and clinical practice. Some classification systems stress aspects of the language disorder or “progressive aphasia” making little reference to behaviour or the term “dementia” (Gorno-Tempini *et al*., 2011). In contrast, others highlight the overlap in behavioural change seen in this condition with frontotemporal dementia, preferring the term “semantic dementia” (Neary *et al*., 1998). Whilst this may represent research interests in different areas of cognition, it also illustrates the challenge that semantic dementia provides in terms of whether to position the condition within the field of aphasia or the field of dementia care and how this influenced practice as a result. As described, therapy strategies in the current literature particularly target the language disorder, with techniques drawn from aphasia therapy. Interestingly, none of the therapists in this study were aiming to improve underlying language functions, such as word finding. A number of therapists reported that experience over time with this client group had shaped their practice. They reported, for example, attempting to teach or maintain a “core vocabulary” when they first worked with this client group, before discontinuing this for a more functional approach, due to concerns that teaching words made relatively little difference to the individual’s communication in everyday settings or, in terms of clinical prioritisation, that there were other, more pressing, tasks to be delivered. Instead, the therapist in this study focused on everyday activity and participation, with underpinning values drawn from models of person centred dementia care (Brooker, 2007). It would appear therefore that research examining approaches from both aphasia therapy and dementia care appear potentially useful to the field of semantic dementia.

## Limitations

The therapists here, whilst very experienced, were working either exclusively within mental health settings or with dedicated sessions to such services in the north of England. The findings, therefore, may reflect the broader values and approach of mental health services. Provision of SLT to dementia services across the UK is patchy and so in some areas this dedicated provision in not available and so referrals may be directed to generic adult SLT’s. This study does not address the priorities of these clinicians and is limited to one area of the country. Further research is required to explore therapy provided in different services with potentially different team structures, philosophical underpinnings and in different geographical locations, nationally and internationally. This study also focused on the perceptions and priorities of therapists and therefore does not address whether this fits with the priorities of people with semantic dementia and their families and research exploring this would be extremely worthwhile.

## Practice and research implications

The most important practice implication from this study is the recommendation to move away from a focus solely on the person with semantic dementia to one also encompassing those around the person. This includes family caregivers but also paid care workers, other disciplines and agencies involved in the assessment and care of the person concerned. Seeking out such contacts requires the therapist to move from a clinic or hospital-based service to a flexible service orientated to the community. In addition, there is a need to focus therapy on individualised communication strategies arising out of the challenges identified by the person and their family within everyday activities, rather than performance in test situations. This is not generic advice-giving but one that is generated from close analysis of the individual case. It is important to remember the progressive nature of semantic dementia and to plan not just in the present but also to consider future change when setting goals. Emotional support, as well as advice about communication, was an important part of these therapists’ work. Therapists actively sought out peer supervision for their activities through clinical education networks beyond their locality. Lastly, a greater dialogue between those in research and those in expert clinical practice roles would also help to design clinically-orientated research studies that explore the priorities of those in clinical practice.

## Figures and Tables

**Table 1:** Aspects of the clinical role for therapists

Aspects of the clinical role in semantic dementia	Number of SLTs
Assessment	6
Individual therapy with people with semantic dementia	6
Therapy involving others (partner, family, friend, other)	6
Case/care review meetings	6
Education/information giving	6
Differential diagnosis	5
Joint sessions with other disciplines	4
Group therapy	2

## Note

1 The practice areas identified in this study fell into several such chapters of the ICF: d: Activities and participation (including Chapter 2 “General tasks and demands”; Chapter 3 “Communication”; Chapter 9 “Community, social and civic life”) and e: Environmental factors (including Chapter 1 “Products and technology”; Chapter 3 “Support and relationships”; Chapter 4 “Attitudes”).
